# Ethics Without Borders

**DOI:** 10.1371/journal.pmed.1000119

**Published:** 2009-07-28

**Authors:** The PLoS Medicine Editors

## Abstract

The *PLoS Medicine* Editors discuss the difficulties encountered by nongovernmental organizations when they conduct research, and how ethical oversight of such research needs to be rigorous, but also pragmatic.

Many nongovernmental organizations (NGOs) provide medical care and humanitarian assistance to some of the most vulnerable populations on earth. Increasingly, such organizations are also important producers of health research, which can range from simple health surveys or interview studies, to complex clinical trials.

There is little doubt that the results of such research can be immensely valuable. First, they may be critical in informing the scale and type of interventions an NGO may need to deliver—for example, a survey of growth among children being treated for malnutrition [1]. Second, they may provide crucial data against which the effects of ongoing events can be monitored—for example, the effect on displaced populations of access to maternal health care [2]. Third, they may be used to inform health policy at the highest levels—for example, studies of anti-malarial efficacy among populations served by NGOs can inform recommendations for treatment regimens internationally (reviewed in [3]).

Data collection at NGOs, however, does not have research per se as its primary aim. Rather, it is usually, and rightly, aimed at improvement of delivery of care to populations who normally lack access to services; it often also provides evidence for advocacy on behalf of these populations. Undoubtedly the tension between research and delivery of care is not easy for NGOs to reconcile, catapulting as it does what are essentially care-providing organizations into a whole different sphere—that of scientific investigation involving human participants. Such endeavors raise new and important issues of oversight. Not least, the procedures necessary for ethical conduct of research [4], above and beyond accepted health care guidelines, may not always fit naturally into the established operations of NGOs. Nonetheless, international ethical standards require adequate oversight whenever a line is crossed from simple delivery of care to asking a research question. Knowing where the line lies is one of the most difficult issues for researchers, organizations, and, increasingly, for journals.

Most would accept the need for the ethical review of randomized clinical trials, and that registration in a clinical trials registry plus proper reporting is best research practice [5]. But what about other types of research—such as the example above of a nutritional survey for the purposes of monitoring the growth of children or a human rights investigation of the health care experiences of drug abusers in detention [6]? We'd argue that if the research is done with the intention of gaining generalizable knowledge or publishable results, rather than performing a routine internal audit, it is by definition research and ethical oversight is needed.

This is not a new concept: the need for oversight in research comes out of the long history of the development of protection of research participants. Such guidelines include the Declaration of Helsinki and the International Ethical Guidelines for Biomedical Research Involving Human Subjects from the Council for International Organizations of Medical Sciences [7], which lay out the four basic principles underlying need for ethical review: respect for persons; beneficence; nonmaleficence; and justice. Interestingly, these guidelines note that “the subjects selected should be the least vulnerable necessary to accomplish the purposes of the research”—a clause that poses immediate issues for NGOs who necessarily work with the most vulnerable populations.

In an article this month in *PLoS Medicine* on the experiences of the Médecins Sans Frontières (MSF) research ethics review board [8], Doris Schopper and colleagues discuss how the board has attempted to define what constitutes research, develop a review process appropriate for the organization, and provide adequate protection for participants in research carried out by MSF. There is no question that the research done by MSF and other humanitarian organizations is done in the most difficult of circumstances—“research on the run” as one of the moderators at a recent United Kingdom MSF research event [9] called it—and that there are practical difficulties in obtaining and providing oversight for these organizations, which simply do not apply in other research contexts. What perhaps lies behind some of the hesitation in applying ethical guidelines to the research that NGOs do is a concern that this oversight may interfere with the practicalities of doing the research—that “there's no time” to get ethical approval, or no time for an appropriate board to be set up. By setting up its own independent board, MSF has gone a long way to fill in for a lack of ethical boards in many of the places where they work. But as Schopper and colleagues describe, they have gone further by establishing procedures for obtaining “emergency” review and approval when time is short.

What should be the role of journals in this process? Journals and editors are primarily facilitators for the dissemination of research. However, they also have a duty to ensure that the research they publish adheres to accepted ethical standards. While journal editors cannot affect work already done, they can support initiatives such as the MSF research ethics board and can continue to require clear documentation, both upon submission and within the published article itself, that the research was conducted ethically and ethical review was sought. Ultimately, by refusing to consider for publication research papers that lack ethical review, journals can promulgate an expectation that organizations incorporate such review into their research plans. To lose important research evidence from NGOs due to lack of appropriate oversight constitutes a tremendous waste of the resources involved in conducting the research, introduces a potential source of bias in the literature, and ultimately betrays the trust of research participants.

NGOs have a long and proud history of caring and speaking for the most vulnerable populations. That such organizations now conduct research to inform their care and advocacy is to be welcomed. Their messages are widely heard; conducting and publishing high-quality research that adheres to the highest principles can only empower their voices further.

## Abbreviations

MSF: Médecins Sans Frontières
NGO: nongovernmental organization
